# Pulmonary Aplasia with Unusual Associations in a Woman

**Published:** 2014-03

**Authors:** Arshad Altaf Bachh, Sridhar Pulluri, Aadil Beigh, Chippa Raju, Ranganath Deshpande

**Affiliations:** Department of Pulmonary Medicine, Mamata Medical College and Hospital, Khammam, Andra Pradesh, India

**Keywords:** Lung agenesis, Renal agenesis, Müllerian anomalies

## Abstract

Failure of development of the primitive lung bud leads to an extremely rare congenital anomaly with a prevalence of 34 per 10 lac live births termed pulmonary aplasia. In half of such cases, associated congenital malformations of the cardiovascular, skeletal, gastrointestinal, or genitourinary systems are present. The Mayer-Rokitansky-Küster-Hauser (MRKH) syndrome is defined as the congenital aplasia of the uterus and the upper two thirds of the vagina with normal secondary sexual characteristics, ovaries, and a normal karyotype (46, XX). We report an extremely rare association of right lung aplasia, MRKH syndrome, and right renal agenesis with left pelvic kidney, which to the best of our knowledge is the first such association reported.

## Introduction

Pulmonary aplasia is an extremely rare congenital anomaly representing the failure of development of the primitive lung bud with a prevalence of 34 per 10 lac live births.^1^ This anomaly was first discovered by De Pozze (1673) accidentally during the autopsy of an adult woman. Muhamed (1923) reported the first case from India at a medico-legal autopsy.^2^ Associated congenital malformations of the cardiovascular, skeletal, gastrointestinal, or genitourinary systems are present in half of the cases.^3^ A history of recurrent chest infections during the first year of life is the usual history elicited; however, the patient may be completely asymptomatic and diagnosed accidentally from the radiograph or detected during autopsy.^2^

The Mayer-Rokitansky-Küster-Hauser (MRKH) syndrome, which occurs in one in 4500 female births, is defined as the congenital aplasia of the uterus and the upper two thirds of the vagina with normal secondary sexual characteristics, ovaries, and a normal karyotype (46, XX).^4^ We report an extremely rare association of right pulmonary aplasia, MRKH syndrome, and right renal agenesis with left pelvic kidney, which to the best of our knowledge is the first such association reported.

## Case Report

A 20-year-old woman presented to our hospital with a history of cough with expectoration for 15 days and fever for 10 days. The patient reported to have had dyspnea on exertion for the previous three years and recurrent chest symptoms in her childhood which were not investigated. The patient, daughter of a consanguineously married couple, had not attained menarche.

Physical examination revealed a young alert woman, without any respiratory distress,  who spoke full sentences. She was moderately built and nourished with height of 150 cm and weight of 55 kg with a body mass index (BMI) of 24.44 kg/m^ 2 ^. General examination demonstrated pallor, fever (temperature of 39.4^o^C), normotension, and oxygen saturation of 97% at room air. There was no cyanosis, clubbing, or peripheral edema. Chest examination showed that the lungs were non-traumatic and asymmetrical with the shift of the mediastinum to the right. Tactile fremitus was absent on the right side with ipsilateral diminution of breath sounds and dullness to percussion. Heart sounds were auscultated over the right hemithorax. The rest of the examination was unremarkable.

**Figure 1:**
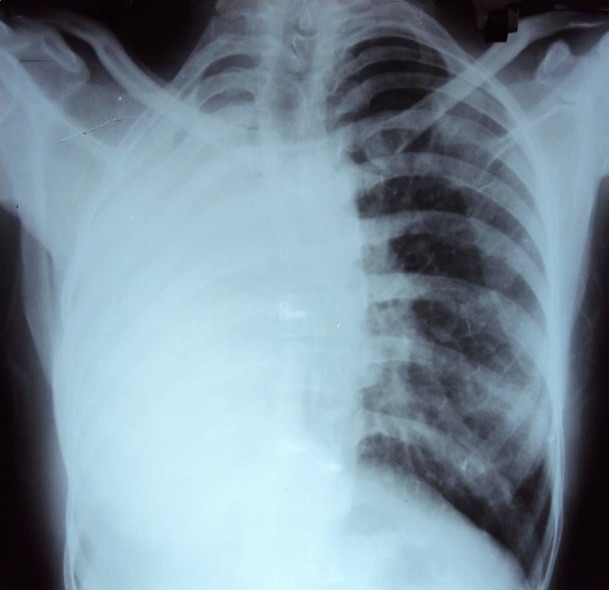
Chest radiography, showing opaque right hemithorax with the crowding of the ribs and mediastinal shift to right.

**Figure 2:**
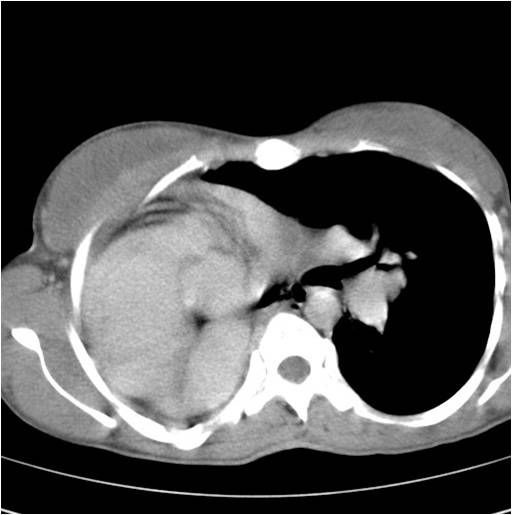
Computed tomography (CT) of the thorax, showing the total absence of the right lung with a blindly-ending right main bronchus, complete shift of the mediastinum to the right, and left lung hyperinflated with herniation to the right hemithorax.

**Figure 3:**
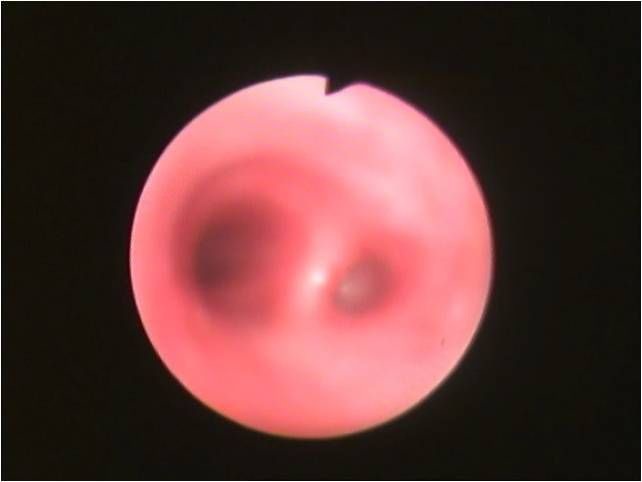
Bronchoscopic view at carina, showing a rudimentary right main bronchus ending in a blind pouch.

Routine blood counts revealed neutrophilic leucocytosis, with total counts of 14000 per dl. Liver functions revealed hypoalbuminemia, and renal functions were normal. Sputum was negative for acid fast bacilli but gram stain showed gram-negative bacilli. Chest roentgenogram showed opaque right hemithorax with the crowding of the ribs and mediastinal shift to right (figure 1). Computed tomography (CT) of the thorax showed total absence of the right lung with a blindly-ending right main bronchus (aplasia), complete shift of the mediastinum to the right with a hyperinflated left lung herniating to the right hemithorax, and a prominent left pulmonary vasculature (figure 2). Fibre optic bronchoscopy revealed a normal left bronchial tree and a right main bronchus ending in a blind pouch (figure 3). Culture of aspirate from rudimentary bronchus grew *Pseudomonas*. An electrocardiogram showed a normal sinus rhythm of 90 beats per min, with a rightward axis. Echocardiography revealed a dilated right heart, with a normal left and right ventricular function, and no valvular abnormality.

**Figure 4:**
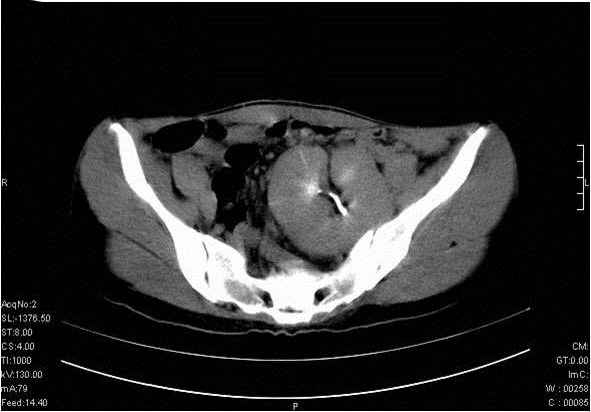
Contrast-enhanced computed tomography of the pelvis, showing left renal pelvicalyceal system superior to the bladder with a short ureter, suggestive of left ectopic (pelvic) kidney.

Gynecologic evaluation for primary amenorrhoea was done. The examination revealed well-developed secondary sexual characteristics. There was partial vaginal atresia and on rectal examination, the pelvis was noted to be free, suggesting a Müllerian abnormality. Transabdominal ultrasonography confirmed the absent uterus, and both kidneys were not visualised in the lumbar regions. A solitary left ectopic kidney, measuring 9.1×6.2 cm, was seen superior to the bladder in the pelvis. CT of the abdomen and pelvis confirmed right renal agenesis with left ectopic (pelvic) kidney and absent uterus (figure 4). Karyotyping verified the 46 XX pattern, thus confirming the MRKH syndrome as a cause of the primary amenorrhea. The final diagnosis was confirmed as congenital right lung aplasia with bronchial stump infection, MRKH syndrome, right renal agenesis, and left pelvic kidney. The patient's respiratory symptoms responded well to a 10-day course of anti-pseudomonal antibiotics and other supportive treatment. She was counselled about the treatment options available to restore her sexual function when she is emotionally mature and ready to start sexual activity.

## Discussion

Schneider classified pulmonary agenesis into three groups, which were later modified by Boyden.^2^ There is a complete absence of lung and bronchus and there is no vascular supply to the affected side in type 1 (agenesis). There is a rudimentary bronchus with the complete absence of pulmonary parenchyma in type 2 (aplasia), and variable amounts of bronchial tree, pulmonary parenchyma, and supporting vasculature are present in type 3 (hypoplasia). Our reported case can be classified as type 2 pulmonary agenesis or aplasia. The associated congenital anomalies are present in half of the cases.^3^ Bronchoscopy can help to identify the rudimentary bronchus to establish a final diagnosis of aplasia lung. Some cases require angiography. The prognosis of cases with aplasia lung varies depending upon the functional ability of the remaining lung and the presence of associated anomalies.^2^

Genital and sometimes extra genital malformations are associated with embryologically unilateral renal agenesis (RA). Different organ malformations of mesodermal origin, such as the heart, lung, and urogenital system, including Müllerian anomalies can occur in combinations.^5^ Mirapeix et al.^6^ reported a rare association of left renal agenesis and left pulmonary hypoplasia in a 46-year-old woman. Kaya and Dilmen^7^ reported a case of right lung agenesis with the absence of the left kidney and fusion anomaly between the fourth and fifth ribs on the left hemithorax. The simultaneous malformations of the lungs and kidneys was suggested to occur because of three reasons: 1) induction of the mesoderm is required on the bronchial and ureteric buds; 2) single teratogen might affect the development of both as they develop during the same period (5^th^ week of gestation); and 3) for the development of lungs, the lung growth factors are produced by the kidney. Acién et al.^5^ reported the association of renal dysplasia, pulmonary hypoplasia, and MRKH Syndrome in a 17-year-old woman. To date, the association of renal agenesis, pulmonary aplasia, and MRKH syndrome, as was the case in our patient, has not been reported. The MRKH syndrome is defined as the congenital aplasia of the uterus and the upper two thirds of the vagina with normal secondary sexual characteristics, ovaries, and a normal karyotype (46, XX). Oppelt et al.^8^ classified their 53 cases of the MRKH syndrome in three recognized subtypes: typical, atypical, and MURCS (Mullerian duct aplasia, renal aplasia, and cervicothoracic somite dysplasia).

## Conclusion

Malformations of the renal system were the most frequent type of accompanying malformation, followed by skeletal changes. The MRKH syndrome with lung agenesis is a rare association.
